# Field-ready DNA extraction from scat using magnetic nanoparticles for non-invasive wildlife monitoring

**DOI:** 10.1038/s41598-026-37759-6

**Published:** 2026-01-30

**Authors:** Letizia Dondi, Rahul Chaudhari, Natalie Schmitt, Jocelyn Poissant, Marco Musiani, Carlos D. M. Filipe, Yingfu Li

**Affiliations:** 1 School of Biomedical Engineering, McMaster University, Hamilton, Canada; 2 Department of Chemical Engineering, McMaster University, Hamilton, Canada; 3 Department of Veterinary Medicine, University of Calgary, Calgary, Canada; 4 Department of Biological, Geological, and Environmental Sciences, University of Bologna, Bologna, Italy; 5 Department of Biochemistry and Biomedical Sciences, McMaster University, Hamilton, Canada

**Keywords:** DNA extraction, Scat, Field-based methods, Rapid, Minimal equipment, Biological techniques, Biotechnology, Ecology, Ecology, Zoology

## Abstract

Scat is commonly used to non-invasively monitor the presence of wildlife. Samples are typically collected in the field and transported to a laboratory for downstream DNA analysis. Current gold-standard extraction methods rely on column-based kits that are expensive, labor-intensive, and require specialized instrumentation. Here we present a rapid and low-cost DNA extraction method using silica-coated magnetic beads, tested on our species of interest *- Rangifer tarandus* (a Species at Risk currently monitored in Canada using scat sampling) and extended to other Cervidae species. This portable approach requires only simple equipment, yields stable DNA, and is well suited for polymerase chain reaction (PCR) analysis both in the field and in centralized laboratories. We also designed and validated novel primers specific for *R. tarandus* mitochondrial DNA to enable reliable species identification from scat. In this study, we demonstrate the ease of use, DNA yield, stability and PCR success of our method compared with widely used kits. We further show its applicability across additional *Cervidae* scat, underscoring its transferability to other herbivorous mammals.

**Supplementary Information:**

The online version contains supplementary material available at 10.1038/s41598-026-37759-6.

## Introduction

Scat is an animal product that can serve as a reliable indicator of a species’ presence in a given region. This indirect approach to wildlife monitoring allows scientists to obtain valuable information about individuals, populations and the species as a whole, which is crucial to conservation management efforts^1^. Current field identification of species from scat is typically qualitative, based on distinct physical characteristics of the excrement such as size and shape. In Canada, *Rangifer tarandus* (caribou) is considered a species at risk, and monitoring approaches often involve scat collection. However, the scats of closely related Cervidae species, such as white-tailed deer (*Odocoileus virginianus*), are morphologically similar and may be misidentified. To verify the species of origin, samples must be transported to a laboratory for further processing, making this a time-consuming and resource-intensive task. Because caribou frequently inhabit remote areas, samples are often transported under fluctuating temperatures, which can lead to DNA degradation and reduce the ability to obtain useful information^2^. Although shipping under frozen conditions helps preserve DNA, it is costly. Morphology-based scat collection also poses challenges for sympatric species in other regions, such as carnivores in the *Vulpes* and *Canis* genera. For example, a study in China reported that 88% of scats initially attributed to snow leopard were misidentified, leading to bias and financial strain in conservation research^3^. However misidentification rates vary widely depending on observer training, ranging from 1.2 to 84.6%^4^.

Species monitoring is further complicated in low-resource and remote settings, where samples must often be shipped across international borders to reach testing facilities. This process can be logistically and bureaucratically challenging due to permit requirements. A field-applicable DNA extraction test could enable researchers to confirm species identity directly in the field or generate DNA extracts that are easier to ship internationally while preserving the quality of genetic materials. Unlike most biological animal samples, small volume nucleic acids can be exempt from the Convention on International Trade in Endangered Species of Wild Fauna and Flora (CITES), making DNA extracts more practical for shipment^5,6^.

The gold-standard for scat DNA extraction involves column-based kits or automated instruments, which yield clean DNA solution free from contaminants and inhibitors. However, these methods require extensive plastic consumables, specialized equipment, are costly and time-intensive. Alternative protocols for scat DNA extraction exist, but they often involve long incubation times, high-speed centrifugation, and complex workflow^7^. Due to their complexity, laboratories typically charge high fees per sample. Currently, there are no rapid, commercially available, field-applicable extraction protocols for scat that do not require instrumentation.

Magnetic beads have been used for decades to extract high-quality DNA from diverse sample types^8^. Silica-coated iron beads are particularly effective because of the strong affinity between silica and nucleic acids, and they can also be produced using straightforward laboratory methods, reducing costs^9^. In addition, these nanoparticles remain stable across a wide range of temperatures, making them suitable for portable extraction. However, existing protocols often rely on hazardous chemicals such as guanidium thiocyanate or guanidium hydrochloride, used for example in Qiagen extraction kits. These guanidine salts enhance DNA binding to silica but pose risks to the users and the environment^10^.

**Fig. 1:**
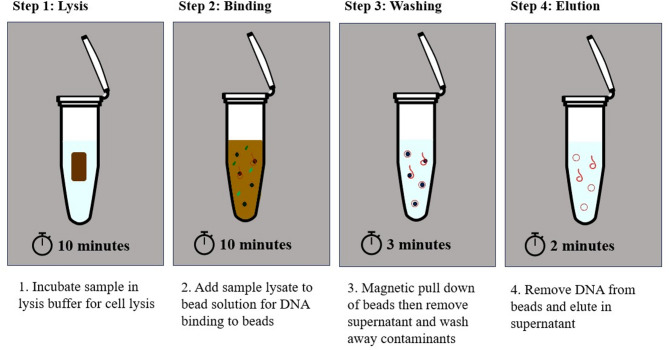
Workflow diagram for the magnetic nanoparticle-based DNA extraction method for *Cervidae* scat.

In this study, we demonstrate the use of magnetic nanoparticles (MNPs) for the rapid DNA extraction from *R. tarandus* scat samples in under 25 min (Fig. 1). We compare DNA yield from our protocol with the QIAamp Fast DNA Stool Kit, the current gold standard, and assess its ability to recover DNA from the scat of other Cervidae species. This method is suitable for fast, instrument-free nucleic acid extraction in laboratory and field settings. It saves time and cost, reduces reliance on plastic consumables, and generates DNA that remains stable for at least seven days at room temperature, enabling downstream PCR analysis. We further describe two one-tube real-time PCR (qPCR) assays: one for the specific amplification of *R. tarandus*, and another for Cervidae detection more broadly. These assays confirm that DNA extracted with our method is PCR-amplifiable and provide conservation biologists with a rapid tool for species-level identification on a single instrument. We anticipate that this approach can be tailored to other species of interest, enabling field-based DNA extraction from scat samples to support global conservation efforts.

## Results

### In-house MNP method optimization

**Fig. 2:**
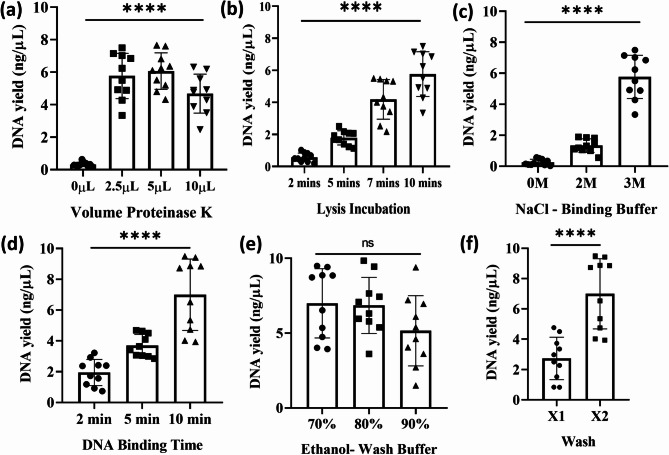
Optimization of in-house MNP DNA extraction protocol. (**a**) Proteinase K concentration in lysis buffer (*n* = 10 technical replicates); (**b**) lysis incubation time (*n* = 10 technical replicates); (**c**) sodium chloride concentration in binding buffer (*n* = 10 technical replicates); (**d**) DNA binding incubation time (*n* = 10 technical replicates); (**e**) percentage of ethanol in wash buffer (*n* = 10 technical replicates); (**f**) number of wash steps (*n* = 10 technical replicates). DNA yield is expressed as ng/µL. Statistical analysis was performed using One-way ANOVA (**a**–**e**) and *t-*test (**f**); **** *P* < 0.0001; ns = no significant difference.

Intending to create a field-applicable DNA extraction method, we focused our efforts on devising a protocol with three key attributes: non-toxic, inexpensive and user-friendly. Scat samples have a high degree of variability in size and shape; therefore, to reduce the variability, *R. tarandus* scat was homogenized, and this homogenate was used to create aliquots for optimization experiments. Sample aliquots contained roughly 100 mg of scat. Using homogenized scat provided technical replicates for protocol optimization. Two replicate groups (*n* = 10 technical replicate) with the same extraction condition were used instead of identical multiple extractions specifically in Fig. 2a (2.5 µL proteinase K volume), Fig. 2b (10 min lysis) and Fig. 2c (NaCl 3 M), and Fig. 2d (10 min binding), Fig. 2e (70% Ethanol) and Fig. 2f (2x wash). Multiple parameters were tested in developing our in-house MNP method, including lysis buffer composition, incubation time, and wash buffer concentrations (Fig. 2).

The first developmental priority was to create a method free of toxic chemicals, improving both user safety and environmental disposal, while yielding sufficient nucleic acid for direct PCR. The in-house lysis buffer conditions were optimized to generate a non-toxic solution. When proteinase K concentrations were titrated in the lysis buffer, we found no significant difference in nucleic acid yield among increasing concentrations within the tested timeframe (Fig. 2a). Unsurprisingly, omission of proteinase K significantly reduced the DNA yield and prevented PCR amplification (One-way ANOVA, F(3,36) = 60.69, *P* < 0.0001; Fig. 2a). To minimize exposure to proteinase K, which is considered an irritation, we opted for 2.5 µL for the lysis buffer^11^. Lysis incubation time was also examined: samples incubated for 10 min yielded an average DNA concentration of 5 ng/µl (One-way ANOVA, F(3, 36) = 58.73, *P* < 0.0001; Fig. 2b). We also considered the herbivorous diet of *R. tarandus* and sought to minimize carbohydrate and polyphenol carryover. Polyvinylpyrrolidone (MW 40,000; PVP-40), commonly used in plant DNA extraction (Cetyltrimethylammonium Bromide method) to remove polyphenols, was therefore incorporated into our buffer^12^.

Commercial DNA extraction kits typically employ guanidium hydrochloride to denature proteins and facilitate DNA binding to silica surface (e.g., Qiagen kits). To avoid guanidine salts due to their toxicity, our assay instead promoted DNA binding by saturating sodium chloride (NaCl) concentrations to reduce hydrophilicity and dissociate DNA–protein complexes. Increasing NaCl concentration significantly enhanced DNA yield (One-way ANOVA, F(2, 27) = 117.5, *P* < 0.0001; Fig. 2c), but the concentrations above saturation caused salt carryover into the elution step, preventing DNA release from the silica-coated MNPs (data not shown). DNA binding to silica also appeared time-dependent, with the highest yield obtained after 10 min of incubation (One-way ANOVA, F(2, 27) = 29.76, *P* < 0.0001; Fig. 2d).

Wash buffer composition was further optimized (Fig. 2e,f). Wash buffers with higher ethanol concentration yielded lower DNA recovery, but this difference was not statistically significant (One way ANOVA, F(2, 27) = 2.175, *P* = 0.1331). This prompted us to use a 70% ethanol wash buffer, as it would reduce overall amount of ethanol and therefore downstream cost. In addition, our binding buffer contained high molarity NaCl to promote DNA-silica binding, washing was essential to dilute residue salts. Wash buffers with reduced water content (i.e. higher ethanol concentration) were less effective at salt removal, maintaining high ionic strength and reducing DNA elution efficiency. This explains why two sequential washes of 70% ethanol produced significantly higher DNA yields than a single wash with 70% ethanol (*t-*test, t = 4.984, df = 18, *P* < 0.0001; Fig. 2f).

The final optimized protocol consisted of the following: lysis buffer (500 µL) containing 1% SDS, 20 mM Tris (pH 8.0), 100 mM NaCl, 2.5 mM EDTA, 2.5 µl of proteinase K (10 mg/ml) and 2% (wt/vol) PVP-40; binding solution (510 µL) containing 100 µL of 2.5 mg/mL PuroMAG Silica-Coated Magnetic Beads, 240 µL of ethanol and 160 µL of 3 M NaCl and 10 µL of PEG 10,000 (50% wt/vl); and wash buffer composed of 10 mM Tris (pH 8.0) in 70% ethanol. We also tested whether our method could extract higher amounts of DNA by increasing the weight of scat in lysate from 100 mg to 150 mg, 300 mg and 450 mg, and saw a proportional increase in DNA yield (One way ANOVA, F(2, 12) = 54.50, *P* < 0.0001; Supplementary Fig. 1).

### Validation of PCR

To determine whether our method extracted DNA suitable for PCR amplification, we searched the literature for *Rangifer tarandus*-specific primers. Only one primer set was identified from Kim and colleagues; however, it lacked specificity because it also produced DNA amplicons of similar sizes in non-targeted species^13^. Therefore, we designed new *R. tarandus*-specific primers (Dl) by aligning mitochondrial sequences from closely related Cervidae species in Canada.

The D1 primer pair was tested for sensitivity and specificity using tissue DNA from *R. tarandus*, *O. virginianus*, *Cervus canadensis* and *Alces alces*. As a positive control to demonstrate DNA extraction from non-*R. tarandus* samples, previously described primers targeting a conserved fragment in all species were used (referred to as QF/R)^13^.

**Fig. 3:**
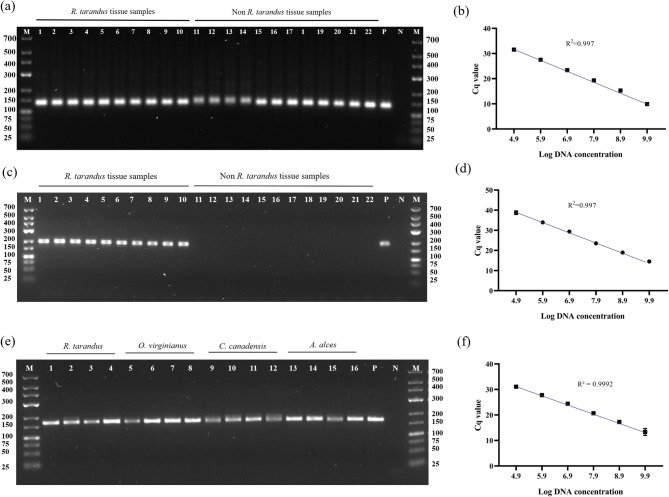
qPCR assay optimization for Dl primer set specific for *R. tarandus* and QF/R primer set for conserved *Cervidae* target. (**a**) Gel electrophoresis analysis of QF/R assay using tissue DNA: Lane 1–10, *R. tarandus*; lane 11–14, *O. virginianus*; lane 15–18, *C. canadensis*; lane 19–22, *A. alces*; P, *R. tarandus* positive control; N, negative control; M, GeneRuler DNA Ladder, Low Range. (**b**) Standard curve of *R. tarandus* DNA with QF/R assay. (**c**) Gel electrophoresis of *R. tarandus* DNA using Dl assay using tissue DNA: Lane 1–10, *R. tarandus*; lane 11–14, *O. virginianus*; lane 15–18, *C. canadensis*; lane 19–22, *A. alces*; P, *R. tarandus* positive control; N, negative control; M, GeneRuler DNA Ladder, Low Range. (**d**) Standard curve of *R. tarandus* DNA with Dl assay. (**e**) Gel electrophoresis analysis of 18 S rRNA nuclear assay using tissue DNA: Lane 1–4, *R. tarandus*; lane 5–8, *O. virginianus*; lane 9–12, *C. canadensis;* lane 13–16, *A. alces*; P, R. tarandus positive control; N, negative control; M, GeneRuler DNA Ladder, Low Range. (**f**) Standard curve of *R. tarandus* DNA with 18 S rRNA nuclear assay.

As seen in Fig. 3a, the qPCR QF/R assay amplified a 137-bp fragment in all tested tissue samples. Optimization of the new D1 primer set with *R. tarandus* tissue confirmed amplification of a 194-bp fragment (Fig. 3c). To determine the limit of detection, a serial dilution of *R. tarandus* DNA was analyzed. Both assays exhibited a limit of detection of 800 fg and an amplification efficiency of 99.7% (Fig. 3b, d).

Furthermore, we developed a nuclear assay to amplify the 18 S rRNA region of all Cervidae species tested. This assay primer set will be denoted throughout the paper as 18 S and amplifies a 164-bp fragment in all tested tissues samples (Fig. 3e). The 18 S assay exhibited a limit of detection of 800 fg and an amplification efficiency of 99.92% (Fig. 3f).

### Comparison to QIAamp fast DNA stool extraction kit

To validate the efficiency of our method against the gold-standard Qiagen kits, we extracted DNA from 100 mg of *Rangifer tarandus* scat using the QIAamp Fast DNA Stool Kit according to the manufacturer’s protocol. A total of 50 scat samples were collected, and two aliquots were prepared from each: one processed with the Qiagen kit and the other with our MNP method. Following extraction, DNA was quantified and analyzed on a 1% agarose gel to assess shearing and fragmentation (Supplementary Fig. 2).

**Fig. 4:**
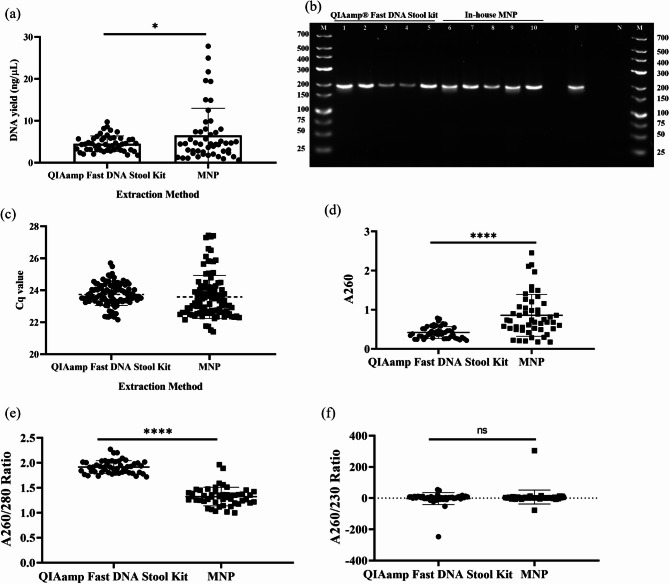
Comparison of DNA extraction from *R. tarandus* scat using the in-house MNP method and QIAamp Fast DNA Stool Kit. (**a**) DNA yield (ng/µL); statistical analysis by *t*-test, t = 2.114, df = 98, *P* = 0.0370 (*n* = 50 biological replicates). (**b**) Gel electrophoresis of qPCR Dl assay products: lane 1–5, *R tarandus* extractions with QIAamp Fast DNA Stool Kit; lane 6–10, *R tarandus* extractions with in-house MNP method; P, positive control; N, negative control; M, GeneRuler DNA Ladder, Low Range. (**c**) qPCR Dl assay Ct values of extracted samples (*n* = 50 biological replicates). (**d**) A260; statistical analysis *t-*test, t = 5.594, df = 98, *P* < 0.0001 (*n* = 50 biological replicates). (**e**) A260/280 Ratio, statistical analysis *t-*test, t = 18.51, df = 98, *P* < 0.0001 (*n* = 50 biological replicates). (**f**) A260/230 Ratio, statistical analysis *t-*test, t = 1.095, df = 98, *P* = 0.2763 (*n* = 50 biological replicates). * *P* < 0.05; **** *P* < 0.0001; ns = no significant difference.

Our in-house MNP method yielded a mean DNA concentration of 6.538 ng/µl (95% CI: 4.708–8.368), compared with 4.538 ng/µl (95% CI: 4.011–5.060) obtained using QIAamp kit (Fig. 4a). Thus, our rapid method extracted significantly more DNA from *R. tarandus* scat (*t*-test, t = 2.114, df = 98, *P* = 0.0370), and the DNA was directly used for qPCR amplification (Fig. 4b, c). As demonstrated in Fig. 4c, scat extracts from both methods produced similar Cq values (~ 23) with the Dl assay. This indicates that target DNA abundance was comparable even when total DNA yield differed between methods.

To estimate the host DNA content, the Cq values were applied to the linear equation derived from the standard curve. We estimated that 100 mg scat samples extracted by either method contained approximately 220 pg of host mitochondrial DNA, representing 3% of the total DNA.

To assess the carryover of inhibitors in the scat sample extracted, we assessed the absorbance of the 50 biological replicates using QIAamp Fast DNA Stool Kit and our MNP method (Fig. 4d–f). As can be seen in Fig. 4e, the A260/280 of our MNP appears to be significantly reduced (*t*-test, t = 18.51, df = 98, *P* < 0.0001) when compared to the column-based method, signifying that some proteins are carried over into the eluted DNA but as this does not impact amplification success. Interestingly, the A260/230 between these method shows no significant difference (*t-*test, t = 1.095, df = 98, *P* = 0.2763), meaning there are salts and other components which absorb at 230, which are carried over in both DNA eluates. This phenomenon has been seen by others when extracting DNA from scat samples^14^.

**Fig. 5:**
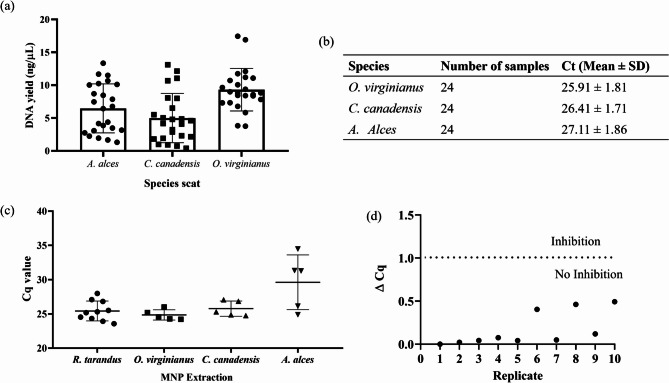
DNA extraction from additional *Cervidae* species using in-house MNP method. (**a**) DNA yield (ng/µL) from scat of *A. alces*, *C. canadensis*, and *O. virginianus* (*n* = 24 biological replicates). (**b**) Quantitative PCR results for QF/R, showing Ct values (mean ± SD) for 24 samples per species. (**c**) qPCR 18 S assay Ct values of extracted MNP samples (*n* = 10 *R. tarandus*, *n* = 5 *O. virginianus*, *n* = 5 *C. canadensis*, *n* = 5 *A. alces*). (**d**) Inhibition spike-in exogenous DNA experiment in MNP extracted *R. tarandus* samples (*n* = 10), calculated Delta Cq value = (mean Ct of spiked exogenous DNA sample replicate) – (mean Ct of exogenous DNA in deionized water). Delta Cq of over 1 signifies inhibitors present in the MNP extract, whereas Delta Cq of under 1 shows no inhibition in qPCR assay.

Using our in-house MNP method, we successfully extracted and amplified mitochondrial DNA from the scat of *O. virginianus*,* C. canadensis* and *A. alces* (Fig. 5a, b). The mean DNA yields were 9.303 (95% CI:7.938–10.67), 4.995 (95% CI: 3.413–6.577) and 6.466 (95% CI: 4.893–8.039), respectively (Fig. 5a). Furthermore, we assessed the amplification of 18 S rRNA nuclear marker for a subset of samples extracted using our MNP method (Fig. 5c). We successfully amplified nuclear DNA from *R. tarandus* (25.42; SD ± 1.455), *O. virginianus* (24.85; SD ± 0.7536) and *C. canadensis* (25.77; SD ± 1.109) however only were able to successfully amplify 2 out of 5 *A. alces* extracts tested as 3 samples exhibited a Ct of over 30 beyond our negative cut-off value (29.61; SD ± 3.994). These are promising results for those looking to extract and amplify nuclear DNA, as this can be done successfully using our MNP method.

Additionally, we tested the inhibitory effect of our MNP samples spiking exogenous synthetic DNA into 10 randomly selected MNP samples. To assess if any inhibitors are carried over the change in Ct between synthetic DNA spiked in MNP extract (*n* = 10 biological replicates) and DNA spiked in deionized water (*n* = 3) was calculated, as Delta Cq (Fig. 5d). If the Delta Cq value, was smaller than 1, there is no inhibition present. As can be seen from Fig. 5d, the spiked DNA samples did not show any inhibitory effect.

**Fig. 6:**
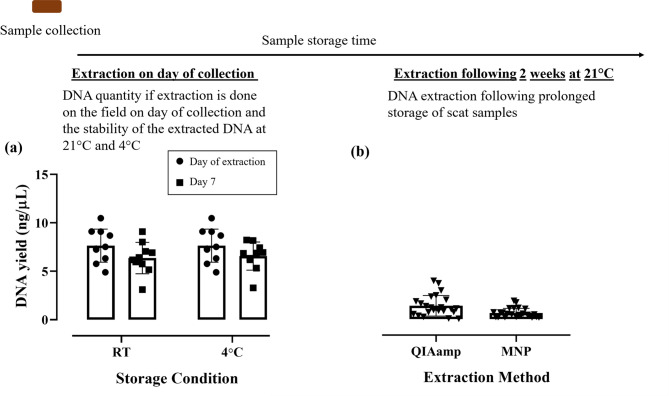
DNA Recovery from fresh scat samples using our in-house method in comparison to prolonged storage of scat (two weeks at room temperature roughly 21 °C) followed by DNA extraction. (**a**) DNA quantification of fresh *R. tarandus* scat samples extracted by our in-house MNP protocol, samples were quantified on the day of extraction and then stored for 7-days at room temperature (RT) or 4 °C. No significant difference was observed (*t-*test, t = 0.2927, df = 18, *P* = 0.7731) (*n* = 10 biological replicates) (**b**) Quantification of DNA extracted using our in-house method against the QIAamp Fast DNA stool kit from samples stored for a prolonged period (two weeks at room temperature roughly 21 °C; *n* = 24 biological replicates).

The phenomenon of DNA degradation in improperly stored or aged samples is documented^15,16^. As shown in Fig. 6a, both extraction methods yielded significantly lower nucleic acid concentrations compared with fresh samples. We tested scat stored at 21 °C for 14 days and observed a marked reduction in DNA yield using both the Qiagen kit and our MNP method (Fig. 6). This result underscores the importance of portable extraction approaches for preserving DNA from field samples. Our method can be applied in the field immediately after collection to recover stable nucleic acid material. To mimic this scenario, we extracted DNA from 10 fresh scat samples with our MNP method and divided each eluate into two storage conditions, assessing the stability after 7 days of incubation. The DNA yield did not differ significantly between day 0 and day 7 (Fig. 6a), and importantly, the DNA remained PCR-amplifiable, producing a positive DI qPCR signal.

Room-temperature storage of intact scat was included as a deliberate stress-testing condition rather than a recommended preservation strategy. Although scat samples are commonly stabilized using ethanol or lysis buffers, exposure to ambient temperatures can occur during field collection or transport, particularly in remote settings. This condition was therefore used to evaluate method performance under degraded-DNA scenarios and to provide a conservative benchmark for extraction efficiency.

To minimize confounding variables and enable a reproducible comparison, scat samples used in the 14-day storage experiment were kept in sealed 1.5 mL tubes under temperature-controlled laboratory conditions (approximately 21 °C), with no intentional exposure to light, moisture, or environmental stressors. This experiment was designed to assess time-dependent DNA degradation under defined conditions rather than to model the full range of environmental variability encountered in field settings. As shown in Fig. 6b, prolonged room-temperature storage resulted in substantial DNA loss regardless of the extraction method used, including the gold-standard kit. Importantly, DNA extracted immediately using the proposed magnetic nanoparticle method remained stable and PCR-amplifiable for at least seven days at room temperature or 4 °C, underscoring the advantage of rapid, field-based extraction to preserve genetic material prior to degradation.

## Discussion

In this study, we describe a rapid DNA extraction method that produces stable nucleic acids in under 25 min, with extracts remaining stable for at least 7 days at 21 °C and 4 °C. The method was designed to minimize reagent toxicity, improving user safety and reducing environmental impact. Its strong performance, simplicity and low toxicological footprint makes this approach well suited for field applications, particularly in conservation biology. Scat provides a valuable, non-invasive sample type that can yield genetic information without directly monitoring animals. However, host DNA recovery from scat is notoriously difficult because of food residues, bacteria, yeast and viruses within the matrix.

The current gold standard for *R. tarandus* scat DNA extraction involves swabbing of the epithelial layer followed by processing with the Qiagen Tissue and Blood kit^17^. Although this method can yield 6.5–28.6 ng/µl, it requires lengthy incubations and multiple processing steps, resulting in a turnaround time of ~ 24 h and limiting its practical use. In contrast, our MNP method produces comparable yields in a fraction of the time (Table 1).

To optimize the extraction method, we removed a portion of the pellet for subsequent lysis. As species DNA is predominantly located on the external surface of scat^17^, our approach yields sufficient DNA for downstream PCR amplification but may include non-target material (digestion biproducts, bacteria and other microorganisms). More experienced users may prefer swabbing the exterior of the scat prior to MNP extraction to enrich for target species cells. Adopting the method described by Ball et al. is expected to reduce total DNA yield while increasing the proportion of target DNA^17^. In our hands, external swabbing was inconsistent and required high manual dexterity; therefore, we present pellet subsampling as a practical and reproducible alternative.

As a consequence of working with bulk scat material, conserved nuclear targets such as 18 S rRNA may amplify DNA from non-target sources, including dietary plant material present in fecal extracts. Accordingly, amplification of the 18 S locus should be interpreted as evidence of recoverable nuclear DNA rather than definitive host DNA. In this workflow, species assignment relies on mitochondrial markers, which provide higher taxonomic specificity and are less susceptible to diet-derived interference. Where host-specific nuclear confirmation is required, amplicon identity should be verified by sequencing or by using species-specific nuclear primers.

**Table 1:** Comparison of QIAamp fast DNA stool kit and in-house MNP DNA extraction protocols for Rangifer Tarandus scat.

Species	Number of samples	Extraction method	Lysis Buffer	Heat	Binding Buffer	Instrumentation	Processing time	Cost per sample	Mean Yield (ng/µl, ±SD; 95% CI)	Mean Cq (± SD)
*Rangifer tarandus*	50	QIAamp Fast DNA Stool Kit	Guanidium hydrochloride, proteinase K	Required	Guanidium hydrochloride, malic acid	Heat Block, Centrifuge	> 2 h	$ 8	4.54 ± 1.85 (95% CI: 4.01–5.06)	23.73 (± 0.69)
*Rangifer tarandus*	50	In-house MNP	SDS, proteinase K	Not required	Sodium chloride	Magnet	< 30 min	< $1	6.54 ± 6.44 (95% CI: 4.71–8.37)	23.55 (± 1.34)

A major challenge in DNA extraction is achieving efficient lysis. Most existing protocols rely on highly toxic chemical reagents or mechanical bead disruption. The advantage of our method lies in avoiding these toxic substances, which pose risks to users and the environment. We acknowledge that omitting these reagents can reduce lysis efficiency, potentially explaining the broader distribution of DNA yields observed in Fig. 4a. Nevertheless, we optimized lysis conditions and demonstrated that our method achieves extraction performance comparable to guanidinium hydrochloride–based protocols, but with less processing time, greater ease of use, and much lower cost. Our method costs roughly $1 per sample compared $8 for commercial column kits, and it reduces reliance on plastic consumables - an important benefit for low-resource field settings. The calculation of this price can be found in our Supplementary Information (Supplementary Tables 1, 2). As our method uses only two 1.5 mL tubes, one for lysis and one for MNP extraction, our plastic consumable waste is reduced in half compared to the QIAamp Fast DNA stool kits (1x lysis tube, 1x filter tube, 3 x tubes for flow-through and elution).

Another critical consideration for rapid methods is compatibility with downstream applications. Aikawa and colleagues developed a simple DNA extraction protocol for sika deer and Japanese serow, but it was incompatible with PCR amplification^18^. Crude extraction methods often retain inhibitors that interfere with PCR, necessitating dilution and reducing sensitivity. For example, chelex-100 extractions require dilution prior to amplification. Similarly, Mason and Botella’s Whatman paper strip method showed inconsistent sensitivity and poor amplification^19^. By contrast, our MNP method does not appear to carry over inhibitors enabling direct PCR without dilution and producing results comparable to Qiagen extracted DNA. Importantly, the extracted material can be used directly in qPCR assays for species identification.

Our approach is also readily scalable. By using deep-well sample plates and commercially available magnetic racks, which can be combined with a 96-deep-well plate or other racks, up to 96 samples can be processed simultaneously, matching the throughput of automated platforms such as the QIAcube HT or KingFisher instruments—but without costly robotics. Scalability is particularly valuable in conservation studies where large numbers of scat samples are collected. We simultaneously extracted DNA from *R. tarandus* scat samples, at Toronto Zoo using a 96 deep-well plate, loaded with lysis solutions, bead solutions, wash buffers and elution buffers to multiplex extraction using a multichannel pipette (data not shown).

We further demonstrated that storage of *R. tarandus* scat at room temperature reduces DNA yields (Fig. 6). However, extracts generated with our method remained stable for at least seven days, preserving PCR-amplifiable DNA. This capability allows researchers to perform extractions immediately in the field, minimizing DNA degradation, or to generate stable extracts that can be transported to laboratories more easily than intact scat. Beyond animal DNA analysis, the method could be adapted for pathogen detection, environmental DNA recovery, or extraction from other sample types. The 18 S assays were conduced 2 years after the initial DNA extraction, during which period samples were stored at -20 °C. We demonstrate that this method can preserve DNA which is amplifiable years following initial extraction stored at -20 °C.

One key limitation of our study is that we did not evaluate variability in scat samples stored under field conditions for extended periods or exposed to high-temperature environments^16,20^. Such conditions may compromise DNA integrity through accelerated degradation, potentially reducing DNA yield and extraction efficiency. Because these factors were not assessed, we acknowledge that inherent DNA degradation in field-exposed samples may influence extraction success and downstream PCR analyses.

Caribou was selected for our study for three reasons: (1) scat represents a complex and challenging matrix for DNA extraction; (2) scat collection is a common practice in conservation monitoring, and (3) Canadian and Albertan governments, in collaboration with industry, are actively monitoring threatened caribou populations but face high costs and logistical burdens with existing protocols.

However, the value of this method extends well beyond caribou. It can be applied to the scat of other species for rapid identification and DNA recovery, supporting easier transport and analysis. We envision the creation of “lab-in-a-box” field platforms that incorporate this method, enabling affordable and reliable PCR-ready DNA extraction in resource-limited environments. Ultimately, the adaptability, scalability, and low cost of this method make it a practical tool for mammalian scat analysis in conservation biology and beyond.

## Methods

### Sample selection

Closely related Cervidae species were selected due to their geographical distribution and habitats. Initially, good quality samples, tissue and hide, were used to test the assay specificity and sensitivity. These samples were supplied by the University of Calgary. The scat from the following tracked live species were obtained from Parks Canada in Jasper, Banff National parks and Nosehill park (Calgary, Alberta) for the purpose of this study; *O. virginianus*,* C. canadensis*, and *R. tarandus*. Research was conducted under research permits of Governments of British Columbia and Alberta, Parks Canada and University of Calgary. All experimental protocols were approved by the University of Calgary Animal Care Committee Studies AC16-0195 and AC20-0110. All methods were carried out in accordance with the relevant guidelines and regulations. Furthermore, all methods are reported in accordance with the ARRIVE guidelines (https://arriveguidelines.org). The Toronto Zoo supplied faecal pellets for the remaining species *A. alces* and *R. tarandus*. Sample collection was approved by the Toronto Zoo Animal Welfare Committee.

### DNA extraction

Extraction of Cervidae tissues and hide has been executed using the DNeasy Blood & Tissue Kits (Qiagen, Cat No. 69504). Pellets were extracted by QIAamp Fast DNA Stool Mini Kit (50) (Qiagen, Cat No. 51604). Rapid DNA extraction from faecal pellets was conducted using silica-coated magnetic nanoparticles. The lysis solution consists of 1% sodium dodecyl sulfate (SDS) Grade Ultra Pure (wt/vol) (Bioshop, Cat No. 151-21-3), 20 mM Tris (pH 8.0) (Bioshop, Cat No. 77-86-1), 100 mM Sodium Chloride (NaCl) (Bioshop, Cat No. SOD004.1), 2.5 mM Ethylenediaminetetraacetic acid (EDTA) (Bioshop, Cat No. EDT001.1) and 2% (wt/vol) Polyvinylpyrrolidone MW 40,000 (PVP-40) (Sigma Aldrich, Cat No. PVP40-50G). The magnetic nanoparticle binding solution is composed of 100 µL of 2.5 mg/mL PuroMAG Silica-Coated Magnetic Beads (SKU: NMG-101), 240 µL of ethanol and 160 µL of 3 M NaCl and 10 µL of Polyethylene glycol (PEG) 10,000 (50% wt/vl) (Sigma Aldrich, Cat No. 92897-250G-F). The wash buffer is composed of 10 mM Tris (pH 8.0) (Bioshop, Cat No. TRS001) in 70% ethanol. The DNA in our protocol was eluted in 200 µL of TE buffer. Approximately 100 mg of scat was lysed for 10 min in 200 µL lysis buffer with 2.5 µL (50 µg) Proteinase K (20 mg/mL; Thermo Scientific, EO0491), added to the buffer before the sample. Following lysis, the buffer is pipetted into 510 µL of binding solution and incubated for 10 min to allow the DNA to bind the magnetic beads. The tube is then placed on a magnetic rack for magnetic separation; the solution is removed without disturbing the magnet pellet and washed with 500 µL of wash buffer (70% ethanol in TE buffer). Subsequently, the wash buffer is removed without disturbing the pellet and 200 µL of TE buffer is added. The tube is removed from the magnetic rack for DNA elution for 2 min. The tube is then returned to the rack and the eluate is transferred to a clean tube for storage or downstream analysis.

### Real-time qPCR

For qPCR amplification, sequences from the mitochondrial D-loop region were amplified, QF (5’-ATA TTA TGT ATA ATA GTA CAT TAA ATT ATA TGC CCC ATG CTT-3’) and QR (5’- CGC ATG TTG ACA AGA AAG GAT TTG A-3’) were used to amplify DNA from all species of interest^13^. Additionally, we designed a primer pair specific to *R. tarandus* mitochondrial DNA, Dl_15588_fwd (5’- TTT TAT AAA CGT ACA TAT ATG GTC CTG TAC − 3’) and Dl_15829_rvs (5’ - TTG GCG AGA AAG GAT TTG ACT TAA TGT GCT ATG − 3’). The primers were created by aligning the mitochondrial reference sequences of *R. tarandus*, *O. virginianus*, *Cervus canadensis* and *Alces alces* retrieved on NCBI and aligned on MEGA 11. The nuclear assay, consisted in amplifying the 18 S rRNA region in all tested species of 164-bp, using primers; 18SCONF1 (5’-TGC CAG TAG TCA TAT GCT TGT CTC AAA GAT TAA-3’) and 18SCONR1 (5’-GCA TGT ATT AGC TCT AGA ATT ACC ACG GTT AT-3’).The qPCR reactions were set-up in a final volume of 10 µL: 5 µL of Luna Universal qPCR buffer (New England Biolabs, Cat No. M3003L) and 250 nM of forward and reverse primers. The volume of DNA extract used in each reaction was 1 µL. The thermocycling parameters were an initial denaturation at 95 °C for 5 min, followed by 40 cycles of denaturing at 95 °C for 15 s and extension at 60 °C for 1 min. Each sample was run in duplicate in each qPCR amplification.The negative cut-off for our qPCR assays is 30Ct, any amplification beyond this point should be ran on agarose gel for verification as primer dimers can generate non-specific signal in qPCR using intercalating dyes.

### DNA quantification and purity

Nucleic acid extracts were quantified using Qubit dsDNA High Sensitivity assay kit (ThermoFisher, Cat number: Q32854) on Tecan instrument (Infinite 200 PRO) to a final volume of 100 µL, composed of 95 µL of dsDNA HS Reagent and Buffer and 5 µL DNA extract. The fragmentation of the DNA was examined on 1% agarose gel using 1X SYBR Safe (Invitrogen, Cat number: S33102) staining. The absorbance of *R. tarandus* scat samples was measured using spectrophotometer DeNovix (DS-11+), using the double-stranded DNA setting and 1µL of sample was loaded for each sample analysed. The following values were used for this analysis, A260, Ratio A260/A280 and Ratio A260/A230.

### Inhibition experiment

To verify if inhibition is present in our MNP extracted samples, an exogenous DNA sample was spiked into 10 randomly selected MNP *R. tarandus* scat extracts. The dsDNA length of the exogenously spiked DNA is 214-bp and the primer set used was ExoF1 (5’ - GTGGCTCTGCAGTGGTTTGACC − 3’) and ExoR1 (5’-CTCCTGACGTAGAAGCGAGGCG-3’). The cycling conditions an initial denaturation at 95 °C for 10 min, followed by 40 cycles of denaturing at 95 °C for 15 s and extension at 58 °C for 1 min. The exogenous DNA was spiked at a concentration of 1 pg/µL in water for positive control (triplicate), and 1 pg/µL for the 10 randomly selected MNP scat extracts tested in duplicate. The inhibitory effect was calculated by Delta Cq value = (mean Ct of spiked exogenous DNA sample replicate) – (mean Ct of exogenous DNA in deionized water). A Delta Cq of over 1 signifies inhibitors present in the MNP extract, whereas Delta Cq of under 1 shows no inhibition in qPCR assay.

### DNA longevity experiments

Ten paired scat sample aliquots from *R. tarandus* were extracted using the Qiagen kit and our MNP method. These extracts were subjected to different temperatures, namely 4 °C and room temperature (21 °C), for multiple days (0 and 7 days) to assess the suitability of this method of preserving DNA in various conditions. Following each time point, the DNA was quantified using the Qubit dsDNA High Sensitivity assay kit on a Tecan instrument. The DNA extracted from scat samples from day 0 was stored at -20 °C until the end of the incubation period. The samples were processed on the same plate for qPCR amplification, and each sample was tested in duplicate.

## Supplementary Information

Below is the link to the electronic supplementary material.

Supplementary Material 1

## Data Availability

All data supporting the findings of this study are available within the paper and its Supplementary Information. Primer sequences and steps for this protocol are detailed in the methods section. As well the data that support the findings of this study are available from the corresponding author.
