# Supplementary material for: Field-ready DNA extraction from scat using magnetic nanoparticles for non-invasive wildlife monitoring
Source: Sci Rep. 2026 Jan 30;16:6733. doi: 10.1038/s41598-026-37759-6 (PMC12913957; doi:10.1038/s41598-026-37759-6)
Supplement: Supplementary file 1 — Supplementary Material 1 [file 41598_2026_37759_MOESM1_ESM.docx]

**Field-Ready DNA Extraction from Scat Using Magnetic Nanoparticles for Non-Invasive Wildlife Monitoring**

Letizia Dondi, Rahul Chaudhari, Natalie Schmitt, Jocelyn Poissant, Marco Musiani, Carlos Filipe and Yingfu Li*

**Supplementary Information**

**Supplementary Fig. 1.** DNA Recovery from different starting amount of scat material. DNA yield increased with greater initial scat weight (150 mg, 300 mg, 450 mg), indicating that the method is scalable and suitable for extracting higher amounts of DNA when larger sample inputs are used. (n=5 technical replicates, one-way ANOVA F (2, 12) = 54.50, P<0.0001)


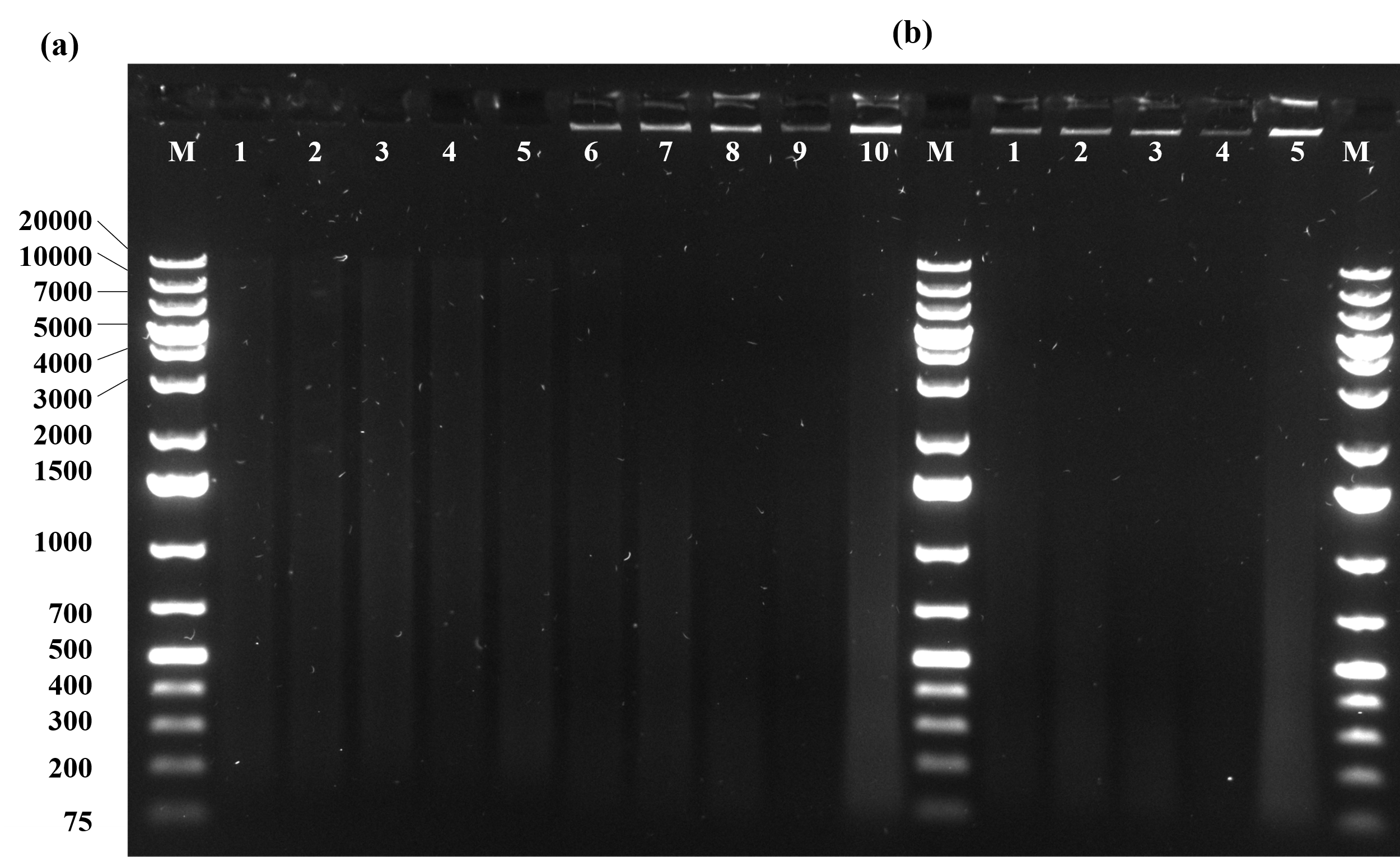


**Supplementary Fig 2.** DNA fragmentation analysis of R. tarandus scat by gel electrophoresis. (**a**) DNA extracted using the QIAamp Fast DNA Stool Kit (lanes 1–5) and the in-house MNP method (lanes 6–10). M: GeneRuler 1 kb Plus DNA Ladder. (**b**) In-house MNP extracts incubated with RNase A to remove RNA contamination (lanes 1–5). M: GeneRuler 1 kb Plus DNA Ladder.

**Supplementary Table 1.** Price calculation of MNP method

| **Component** | **Amount used** | **Unit price** | **Cost (CAD)** |
| --- | --- | --- | --- |
| **Lysis Buffer (500µL)** | | | |
| SDS | 0.002 g | $0.23 / g | $0.00046 |
| PVP-40 | 0.004 g | $1.196 / g | $0.00478 |
| NaCl | 0.00117 g | $0.023 / g | $0.00003 |
| Tris | 0.000485 g | $0.091 / g | $0.00004 |
| **Total lysis buffer** |  |  | **$0.00531** |
| **Bead Solution (510µL)** | | | |
| Magnetic beads | 100 µL | $0.002083333 / µL | $0.20833 |
| Ethanol | 0.24 mL | $0.00953 / mL | $0.00229 |
| NaCl | 0.028 g | $0.023 / g | $0.00064 |
| PEG | 0.005 g | $0.3072 / g | $0.00154 |
| **Total bead solution** |  |  | **$0.21280** |
| **Wash buffer (500µL)** | | | |
| Tris | 0.000606 g | $0.091 / g | $0.00006 |
| Ethanol | 0.35 mL | $0.00953 / mL | $0.00334 |
|  |  | 1X wash | **$0.00340** |
| **Total wash buffer** |  | 2X wash | 0.0068 |
| **Elution buffer (200µL)** | | | |
| Tris | 0.000242 g | $0.091 / g | $0.00002 |
| EDTA | 0.000074 g | $0.064 / g | $0.000005 |
| **Total elution buffer** |  |  | **$0.00003** |

**Supplementary Table 2.** Price calculation of MNP method- buffers and consumables breakdown

| **Material** | **Cost (CAD)** |
| --- | --- |
| Lysis buffer | $0.00531 |
| Bead solution | $0.21280 |
| Wash buffer | $0.0068 |
| Elution buffer | $0.00003 |
| **Total reagents** | **$0.22494 per sample** |
|  |  |
| 1.5 mL tubes (×4) | $0.14155 |
| 10 mL conical (×2) | $0.43200 |
| P1000 tips (×5) | $0.22625 |
| P200 tips (×2) | $0.07141 |
| **Total consumables** | **$0.87121 per sample** |
|  |  |
| **Total per sample** | **$1.09615** |
